# Modeling the probability of a batter/pitcher matchup event: A Bayesian approach

**DOI:** 10.1371/journal.pone.0204874

**Published:** 2018-10-17

**Authors:** Woojin Doo, Heeyoung Kim

**Affiliations:** Department of Industrial and Systems Engineering, Korea Advanced Institute of Science and Technology (KAIST), Daejeon, Republic of Korea; Southwest University, CHINA

## Abstract

We develop a Bayesian hierarchical log5 model to predict the probability of a particular batter/pitcher matchup event in baseball by extending the log5 model which is widely used for describing matchup events. The log5 model is simple and intuitive with fixed coefficients but less flexible than the generalized log5 model that allows the estimation of coefficients using data. Meanwhile, although the generalized log5 model is more flexible, the estimation of coefficients often suffers from a lack of data as a large sample of previous outcomes for a particular batter/pitcher matchup is rarely available in practice. The proposed Bayesian hierarchical log5 model retains the advantages of both models while complementing their disadvantages by estimating the unknown coefficients as in the generalized log5 model, but by using the fixed coefficients of the standard log5 model as prior knowledge. By combining the ideas of the two previous models, the proposed model can estimate the probability of a particular matchup event using a small amount of historical data of the players. Furthermore, we show that the Bayesian hierarchical log5 model achieves better predictive performance than the standard log5 model and the generalized log5 model using a real data example. We further extend the proposed model by including a new variable representing the defensive ability of the pitcher’s team and show that the extended model can further improve the predictive performance of the Bayesian hierarchical log5 model.

## 1 Introduction

Baseball is a sport that requires various strategic data analyses for effective team management; these analyses are known as sabermetrics. Technological developments in data acquisition have accelerated the trend of using historical play data while building game tactics. Baseball managers can use players’ data to optimize the team’s line up and build an appropriate plan for the game. In addition, performance and skill evaluations can help players improve their personal competence and contribute to their team. For example, Oakland Athletics went into the major league baseball (MLB) postseason from 2000 to 2003 despite having the lowest payroll among the major league teams. This was achieved by rebuilding the team with players who could contribute to the team’s victory regardless of their fame or salary. Oakland Athletics’ analyses determined that batter’s on base percentage (OBP) and slugging average (SLG) are more closely related to a team’s victory than runs batted in (RBI), which was regarded as a good indicator of a batter’s hitting ability [1]. Presently, many MLB teams employ data scientists, called sabermetricians, to analyze the game with statistical methods [2].

Research on baseball data analysis has mainly focused on predicting the winner of a game. For example, Barry and Hartigan[3] used a generalized choice model to predict the probability of a team winning a particular game. Bukiet et al.[4] used a Markov chain method to find optimal batting orders and the expected number of games a team will win. Yang and Swartz[5] used a two-stage Bayesian model for predicting winners in major league baseball. Lyle[6] used ensemble learning which employs model trees, artificial neural networks, and support vector machine, to predict players’ future performance. Smith[7] used a Markov chain model for predicting the scores and the winning team of major league baseball games.

However, few studies have focused on the analysis of a particular batter/pitcher matchup event, despite its importance in team building, player usage, and strategic game operation [8]. The log5 model proposed by James is the most widely used model for batter/picther matchup analysis [9]. The log5 model predicts the probability of the occurrence of a binary event, such as home run or strikeout, using three variables: the pitcher’s rate of the event, the batter’s rate of the event, and the league average of the event. For example, when predicting the probability of occurrence of a home run for a matchup, if a batter with a higher home run rate than the league average confronts a pitcher with a higher home run rate than the league average, the log5 model predicts that a home run is likely to occur in that matchup. By extending the log5 model, Healey [10] proposed the generalized log5 model for modeling the probability of a strikeout for a batter/pitcher matchup. The generalized log5 model allows for the three variables in the log5 model to have various coefficients that can be estimated through logistic regression, whereas the coefficients are fixed as 1 or -1 in the standard log5 model.

Although the generalized log5 model is more flexible than the standard log5 model, the estimation of its coefficients can be problematic if sufficient samples of previous outcomes for a particular batter/pitcher matchup are not available. However, sufficient data are rarely available for individual players [11]. To overcome this challenge, Healey [10] partitioned batters and pitchers into one of four platoon configurations according to the handedness (i.e., left-handed pitcher vs. left-handed batter, left-handed pitcher vs. right-handed batter, right-handed pitcher vs. left-handed batter, and right-handed pitcher vs. right-handed batter), and then built a separate model for each group using larger samples of players who belong to the same group. Each of the four models can be used to predict the probability of a strikeout when a batter from a particular group faces a pitcher from a particular group. Although this approach can increase the sample size, the expected outcome of a matchup is significantly influenced by the way the platoon is configured [11]. In particular, using the data of other players to predict the outcome of a particular batter/pitcher confrontation could lead to undesirable prediction results. For example, if there is a batter with a low overall batting average but with a relatively high batting average against a certain pitcher, it may be more meaningful to predict the matchup results by using the corresponding records of the two players only, rather than using a larger dataset from the players in the same platoon configuration. This is often the case when particular two players are in rivalry [12].

In this study, we develop a Bayesian hierarchical log5 model to predict the probability of a particular batter/pitcher matchup event using only a small amount of data from the two players concerned. The Bayesian hierarchical log5 model combines the ideas of the standard log5 model and the generalized log5 model. The proposed model allows various coefficients to be estimated from data, similar to the generalized log5 model; however, unlike the generalized log5 model, the proposed model does not require the platoon configuration. Instead, it uses the fixed coefficients of the standard log5 model as prior information. In this way, we can avoid the problem of inaccurate estimation caused by insufficient examples. To illustrate a specific scenario, we particularly address the problem of predicting whether a batter reaches base safely when he faces a particular pitcher using the data of the two players only. Using the proposed model, we derive the posterior predictive distribution of the probability of reaching base for each batter-pitcher pair. We compare the predictive performance of the proposed model with that of the standard log5 model and the generalized log5 model and demonstrate the effectiveness of the proposed model. Furthermore, we show that the opposite on base percentage of a pitcher’s team is significant for the prediction of the probability that an opposite team’s batter reaches base, and extend our model by including a defense index as a new variable.

The remainder of this paper is organized as follows. We first introduce the log5 model and the generalized log5 model, and then develop a Bayesian hierarchical log5 model in Section 2. The proposed model is validated using real data examples in Section 3. We extend the proposed model by incorporating a new variable in Section 4. Finally, we conclude the paper in Section 5.

## 2 Methodology

### 2.1 Review of log5 model and generalized log5 model

We describe the log5 model for the specific problem of predicting the probability of a particular batter reaching base when a particular pitcher is matched. Let *B* be a batter’s on base percentage (OBP) and let *P* be a pitcher’s opposite on base percentage (OOBP) in a league with an OBP average of *L*. OBP describes how often a batter reaches base in a given period, and OOBP represents how often a pitcher let an opposing batter reach base in a given period.

$$OBP=\frac{H+BB+HBP}{AB+BB+HBP+SF},$$

where *H* = Hits, *BB* = Bases On Balls, *HBP* = Hit By Pitches, *AB* = At Bats, and *SF* = Sacrifice Flies. OBP captures a batter’s positive contribution to the scoring process better than a simple batting average by considering not only the batter’s batting ability but also his plate discipline [13].

The probability of a batter reaching base for a given matchup, denoted by *π*, can be represented using the log5 model as follows [9]:

$$\begin{array}{c} \pi =\frac{(BP)/L}{\left(BP)/L+(1-B)(1-P)/(1-L\right)}. \end{array}$$ (1)

Although in this study we consider the event of a batter reaching base, as mentioned in Section 1, the probability of other events occurring from batter and pitcher confrontations can be modeled similarly using Eq (1) by inserting the corresponding values of the batter, pitcher, and league average. From Eq (1), we can see that *B* and *P* have a positive relationship with *π*, whereas *L* has negative relationship with *π*. This is consistent with our intuition that the likelihood of a batter reaching base is higher if the batter’s OBP and the pitcher’s OOBP are higher than the league average. Owing to its interpretability and simplicity, the log5 model has been widely used for describing matchups in sports [14].

Eq (1) can be rewritten as

$$\begin{array}{c} \pi_{o}=\frac{\left(B_{o}P_{o}\right)}{L_{o}}, \end{array}$$ (2)

where *B*_*o*_, *P*_*o*_, *L*_*o*_, and *π*_*o*_ are the odds ratios for *B*, *P*, *L*, and *π*, respectively:

$$\begin{array}{c} B_{o}=\frac{B}{1-B},P_{o}=\frac{P}{1-P},L_{o}=\frac{L}{1-L},\;\text{and}\;\pi_{o}=\frac{\pi }{1-\pi }. \end{array}$$ (3)

By taking the natural log on both sides, Eq (2) can be equivalently represented as follows:

$$\begin{array}{c} \ln\left(\pi_{o}\right)=\ln\left(B_{o}\right)+\ln\left(P_{o}\right)-\ln\left(L_{o}\right). \end{array}$$ (4)

The generalized log5 model is obtained by introducing coefficients for each variable in Eq (4) as follows:

$$\begin{array}{c} \ln\left(\pi_{o}\right)=\beta_{1}\ln\left(B_{o}\right)+\beta_{2}\ln\left(P_{o}\right)+\beta_{3}\ln\left(L_{o}\right). \end{array}$$ (5)

We can see that the log5 model in Eq (4) is a special case of the generalized log5 model when *β*_1_, *β*_2_, and *β*_3_ are 1, 1, and -1, respectively. In the generalized log5 model, the coefficients *β*_1_, *β*_2_, and *β*_3_ are flexibly estimated using data within each platoon configuration. By taking the exponent of both sides of Eq (5) and solving for *π*, the probability of a batter reaching base can be represented using the generalized log5 model as follows:

$$\begin{array}{c} \pi =\frac{1}{1+e^{-S}}, \end{array}$$ (6)

where *S* = *β*_1_ ln(*B*_*o*_) + *β*_2_ ln(*P*_*o*_) − *β*_3_ ln(*L*_*o*_). Healey[10] estimated *β*_1_, *β*_2_, and *β*_3_ in Eq (6) using the constrained logistic regression with the constraint *β*_1_ + *β*_2_ − *β*_3_ = 1, leading to the desirable property that *π* = *L* if *B* = *P* = *L*.

### 2.2 A Bayesian hierarchical log5 model

The log5 model and the generalized log5 model have both advantages and disadvantages. The log5 model is simple and intuitive, and applicable to a small amount of data. However, it is less flexible as the coefficients for ln(*B*_*o*_), ln(*P*_*o*_), and ln(*L*_*o*_) are fixed as 1, 1, and -1, respectively, as in Eq (4). In contrast, the generalized log5 model is more flexible as the coefficients can have various values. However, it requires a sufficient amount of data for accurate estimation of the coefficients. In practice, a large sample of previous outcomes for a particular batter/pitcher matchup is rarely available. To overcome this difficulty, the generalized log5 model uses data from players who share similar characteristics (e.g., handedness). However, distinguishing players with similar characteristics can be arbitrary, and data from other players is not always helpful for the analysis of a particular batter/pitcher matchup, particularly if the batter and pitcher are in rivalry.

To retain the advantages while complementing the disadvantages of the standard log5 model and the generalized log5 model, we develop a Bayesian hierarchical log5 model that combines the ideas of the two models. The proposed model allows various values of coefficients, similar to the generalized log5 model, but only uses a small sample of previous outcomes for a particular batter/pitcher matchup, similar to the standard log5 model. To overcome the problem of insufficient samples for a particular batter/pitcher matchup, the proposed model uses the fixed coefficients of the standard log5 model as prior information for the various coefficients in the generalized log5 model.

More specifically, suppose that we have a total of *n* matchup events for a particular batter and a particular pitcher, and each matchup event is independent of each other. For the *i*th matchup event, we consider a binary response *y*_*i*_ that takes the value 1 if the batter reaches base and takes the value 0 otherwise. We view *y*_*i*_ as a realization of a Bernoulli random variable *Y*_*i*_ that takes the values of 1 and 0 with probabilities *π*_*i*_ and 1 − *π*_*i*_, respectively. That is,

$$\begin{array}{c} P\left(Y_{i}=y_{i}\right)=\pi_{i}^{y_{i}}{\left(1-\pi_{i}\right)}^{1-y_{i}},\;y_{i}\in \left\{0,1\right\}. \end{array}$$ (7)

Let **x**_*i*_ = (ln(*B*_*o*,*i*_), ln(*P*_*o*,*i*_), ln(*L*_*o*,*i*_))^*T*^ denote a vector of the observations for the *i*th sample, where *B*_*o*,*i*_, *P*_*o*,*i*_, and *L*_*o*,*i*_ denote the values of *B*_*o*_, *P*_*o*_, and *L*_*o*_ for the *i*th matchup, respectively. Let *X* be a matrix of predictors, where its *i*th row is $x_{i}^{T}$, let **y** denote an *n* × 1 vector of *y*_*i*_, and let ***β*** = (*β*_1_, *β*_2_, *β*_3_)^*T*^ denote a vector of regression coefficients.

The Bayesian hierarchical log5 model is given by

$$\begin{array}{ccc} & & Y_{i}∼\text{Bernoulli}\left(\pi_{i}\right),\;i=1,\ldots ,n,\\ & & \pi_{i}=\frac{1}{1+\exp(-\beta^{T}x_{i})},\\ & & \beta ∼\text{Normal}(\mu ,\sigma^{2}I),\\ & & \mu ∼\text{Normal}(\mu_{0},\tau^{2}I),\\ & & \sigma^{2}∼\text{Inverse-Gamma}\left(a,b\right), \end{array}$$ (2)

where ***μ***_0_ is set to ***μ***_0_ = (1, 1, −1)^*T*^ according to the coefficients in the standard log5 model. In our numerical study in Section 3, we specify the hyperpriors as follows: *τ*^2^ = 0.5, *a* = 1, and *b* = 1. The posterior distribution over ***β***, ***μ***, and *σ*^2^ is given by

$$\begin{array}{c} p\left(\beta ,\mu ,\sigma^{2}|X,y\right)\propto p\left(y|X,\beta \right)p\left(\beta |\mu ,\sigma^{2}\right)p\left(\mu \right)p\left(\sigma^{2}\right). \end{array}$$ (8)

The predictive distribution, given a new point x_*_, is computed by

$$\begin{array}{c} p\left(y_{*}|y,X,x_{*}\right)=\int p\left(y_{*}|x_{*},\beta ,\mu ,\sigma^{2}\right)p\left(\beta ,\mu ,\sigma^{2}|y,X\right)d\beta \;d\mu \;d\sigma^{2}. \end{array}$$ (9)

The integral of ***β***, ***μ***, and *σ*^2^ in Eq (9) is done numerically using the Markov chain Monte Carlo (MCMC) method. We first generate *M* samples [***β***^(1)^, ***μ***^(1)^, *σ*^2(1)^], …, [***β***^(1)^, ***μ***^(*M*)^, *σ*^2(*M*)^] from the posterior distribution *p*(***β***, ***μ***, *σ*^2^|*X*, **y**) in Eq (8), and then approximate *p*(y_*_|**y**, *X*, x_*_) by

$$\begin{array}{c} p\left(y_{*}|y,X,x_{*}\right)\approx \frac{1}{M}\sum_{i=1}^{M}p\left(y_{*}|x_{*},\beta^{\left(i\right)},\mu^{\left(i\right)},\sigma^{2(i)}\right). \end{array}$$ (10)

To generate samples from the posterior distribution *p*(***β***, ***μ***, *σ*^2^|*X*, **y**), we used slice sampling [15].

## 3 Experiments

### 3.1 Data

In this study, we used the data of regular-season baseball games in the Korea Baseball Organization (KBO) league from April 2008 to October 2017. The KBO league is a professional baseball organization and the largest and most famous sports league in South Korea. To collect data of whether a batter reaches base or not, we crawled the data from a real-time text broadcasting service provided by the Korea portal site “Naver”. The data of OBP of batters, OOBP of pitchers, and league average OBP were collected from the website “www.statiz.co.kr” that provides statistical records of every player in the KBO. To obtain reliable data of OBP and OOBP, we considered only the data of players with at least 350 plate appearances.

### 3.2 Results

To evaluate the performance of the Bayesian hierarchical log5 model, we selected 5 pitchers and 15 batters from the KBO league who consistently participated in games during the considered period. The list of players is presented in Table 1. We considered every possible pair of the selected pitchers and batters, i.e., a total of 75 batter/pitcher matchup combinations. For each batter/pitcher combination, we first use the matchup data from 2008 to 2014 as a training set and predict the probability of the batter reaching base in 2015. Then, the matchup data in 2015 are also added to the training set to predict the probability of the batter reaching base in 2016. In this way, the training data are accumulated gradually to predict the probability of the batter reaching base in the next year, up to the prediction for 2017. When predicting the probability, we used the yearly average of OBP and OOBP for each player as the predictors in our model.

**Table 1:** List of players considered in this study.

**Pitcher**	**Team**	**Innings**	**Handedness**
S. H. Yoon	Samsung Lions	1523.1	Right
S. J. Song	Lotte Giants	1410.2	Right
H. J. Yang	Kia Tigers	1395.0	Left
W. J. Jang	Doosan Bears	1316.1	Left
K. H. Kim	SK Wyverns	1270.1	Left
**Batter**	**Team**	**Plate appearances**	**Handedness**
G. W. Jeoung	Hanwha Eagles	5266	Right
Y. T. Park	LG Twins	5101	Left
A. S. Son	Lotte Giants	4875	Left
D. H. Lee	KT Wiz	4773	Left
J. Choi	SK Wyverns	4769	Right
S. M. Park	NC Dinos	4763	Right
Y. K. Lee	Hanwha Eagles	4498	Left
J. C. Kim	Kia Tigers	4378	Right
J. K. Park	SK Wyverns	4373	Left
S. H. Jung	LG Twins	4354	Right
J. W. Na	Kia Tigers	4288	Right
B. H. Lee	Kia Tigers	4026	Right
J. Y. Lee	KT Wiz	3909	Left
T. G. Kim	Hanwha Eagles	3907	Right
K. M. Kim	SK Wyverns	3744	Right

Our predicted probability is compared with the true probability, which is calculated by dividing the number of plate appearances for which the batter reaches base when confronting the particular pitcher for the given year by the total number of plate appearances for the considered batter/pitcher matchup. We measured the prediction performance using the mean square error (MSE), then computed the average of the MSE over all 225 tested cases (i.e., 3 cases for each of 75 batter/pitcher pairs). To predict the probability, we estimated the parameters in the Bayesian hierarchical log5 model using slice sampling as previously mentioned. We generated a total of 100,000 samples and discarded the first 10,000 samples in a burn-in period. We checked the convergence of the parameters using Geweke’s diagnostic, which tests for equality of the means of the first and last parts of a Markov chain [16].

The performance of the proposed model is compared with that of the standard log5 model and the generalized log5 model. For the generalized log5 model, platoon configuration of players according to their handedness was used as suggested in [10]. Table 2 summarizes the average of MSE with standard errors in parentheses. We can see that the prediction error of the Bayesian hierarchical log5 model is substantially lower than the other competing models. The generalized log5 and standard log5 models showed similar performance. Table 2 also compares the model coefficients. For the Bayesian hierarchical log5 model, we first calculated the posterior means of the coefficients for each batter/pitcher combination, and then took an average of them over all combinations of batter and pitcher, because different coefficients were estimated for different combinations of batter and pitcher. Similarly, the coefficients of the generalized log5 model reported in Table 2 were obtained by averaging the coefficients over all platoon configurations.

**Table 2:** Comparison of the Bayesian hierarchical log5, generalized log5, and log5 models in terms of MSE and the average of estimated coefficients.

	Bayesian Log5	Generalized Log5	Log5
**MSE**	0.0464 (0.1522)	0.0780 (0.1939)	0.0769 (0.1922)
*β*_1_	0.8179	0.6363	1
*β*_2_	0.1693	1.2859	1
*β*_3_	-1.9412	-0.9222	-1

**Fig 1:**
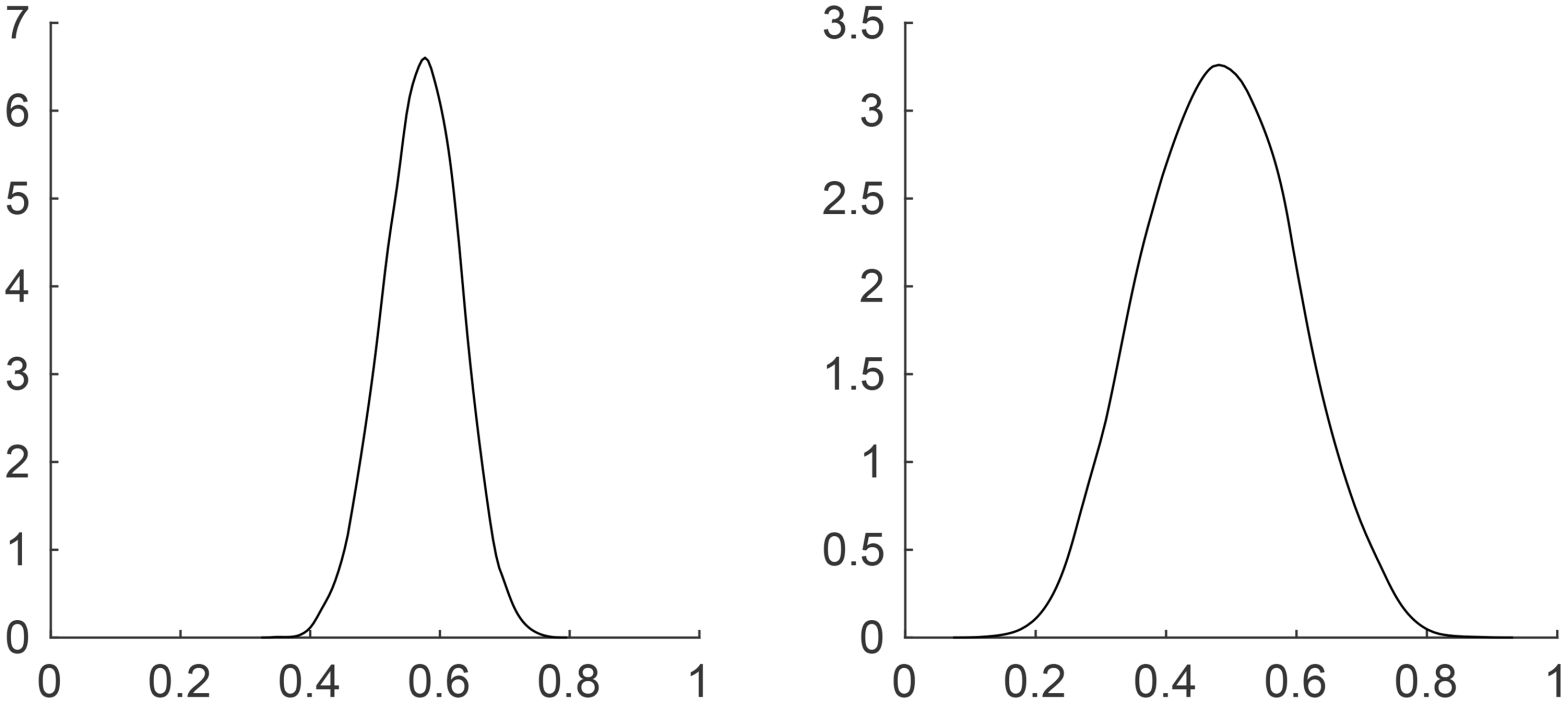
Illustration of the predictive distributions for two different players (Left: J. C. Kim; Right: B. H. Lee).

In addition to higher predictive accuracy, the Bayesian hierarchical log5 model produces a predictive distribution which gives richer information of the probability that a batter reaches base. For example, the left and right panels of Fig 1 show the predictive distributions of the probability of reaching base for two batters, J. C. Kim and B. H. Lee, respectively, when confronting the pitcher K. H. Kim during the 2015 KBO season. It is clear that the predictive distribution for J. C. Kim results in a narrower distribution than B. H. Lee, which indicates that the prediction for J. C. Kim has less uncertainty than the prediction for B. H. Lee. The differently estimated posterior means of the coefficients for J. C. Kim and B. H. Lee were also compared in Table 3.

**Table 3:** Comparison of the estimated coefficients of two different matchups.

	J. C. Kim	B. H. Lee
*β*_1_	0.8198	0.5743
*β*_2_	0.3722	1.8227
*β*_3_	-1.4275	-1.0134

Furthermore, to see if we can improve the predictive performance for a particular pitcher in a certain matchup using data from all other matchups in which he participated, we also considered an extended version of the Bayesian hierarchical log5 model as follows:

$$\begin{array}{ccc} & & Y_{ij}∼\text{Bernoulli}\left(\pi_{ij}\right),\;i=1,\ldots ,b,\;j=1,\ldots ,n_{i},\\ & & \pi_{ij}=\frac{1}{1+\exp(-\beta_{i}^{T}x_{ij})},\\ & & \beta_{i}∼\text{Normal}\left(\mu ,\sigma^{2}I\right),\\ & & \mu ∼\text{Normal}(\mu_{0},\tau^{2}I),\\ & & \sigma^{2}∼\text{Inverse-Gamma}\left(a,b\right), \end{array}$$

where *i* is a batter index, *b* is the number of batters who faced the concerned pitcher, *j* is a matchup index, and *n*_*i*_ is the total number of matchup events between the concerned pitcher and batter *i*. In this model, ***β***_*i*_, *i* = 1, …, *b*, are drawn from a common prior distribution. Using this model, the averaged MSE was obtained as 0.0514 (standard error: 0.1235), which is smaller than that of the standard log5 and generalized log5 models, but is larger than that of the Bayesian hierarchical log5 model in Table 2. These results show that for the prediction of the outcome between a particular pitcher and a particular batter, it may be more effective to use the data of the two players only, rather than to borrow the information from other players.

## 4 Model extension

### 4.1 Four-variable Bayesian hierarchical log5 model

We extend the Bayesian hierarchical log5 model by adding a new variable, which represents a defense index, and suggest a four-variable Bayesian hierarchical log5 model. Naturally, a batter who meets an opponent team with a good defensive ability will have a hard time getting on base. For example, even if a pitcher makes a mistake and allows a ground ball from the batter, a good defensive team can catch the ball quickly and stop the batter from getting on base. Therefore, a pitcher on the mound can throw a more confident and stable pitch if he trusts his team’s defense. Many defensive tactics about keeping batters from getting on base via well-organized plays imply that not only the pitcher’s pitching ability but also the defensive ability of the infield and outfield are important in preventing opponent batters from getting on base.

The relationship between a team’s defensive ability and the rate of opponent batter’s reaching base may be illustrated by investigating how a pitcher’s OOBP changes when they change team (e.g., from a poor defensive team to a good defensive team or from a good defensive team to a poor defensive team). Table 4 shows the changes in OOBP of two pitchers who changed team. W.C. Cha, a pitcher for the Samsung Lions, a team with a poor defense efficiency ratio from 2014 to 2016, recorded the lowest OOBP after transferring to the LG Twins, who had the highest defense efficiency ratio in the KBO league in 2017. E.B. Song, a pitcher for the SK Wyverns, who had top-of-the-league defensive abilities, recorded the highest OOBP after moving to the Kia Tigers, who had the lowest defense efficiency ratio in 2017. These two cases show that whether an opponent batter reaches base or not is highly dependent on the defensive ability of the pitcher’s team.

**Table 4:** OOBP changes of two pitchers according to team.

W.C. Cha			E.B. Song		
Year (Team)	DER (Rank)	OOBP	Year (Team)	DER(Rank)	OOBP
2014 (SS)	0.668 (4)	0.351	2010 (SK)	0.702 (1)	0.307
2015 (SS)	0.679 (3)	0.325	2011 (SK)	0.716 (1)	0.332
2016 (SS)	0.669 (5)	0.365	2012 (SK)	0.703 (3)	0.356
2017 (LG)	0.685 (1)	0.297	2013 (KI)	0.647 (9)	0.416

As a defense index, we use the defensive efficiency ratio (DER), which is a classic sabermetrics measure used to evaluate team defense ability rather than a certain player’s defense ability. The DER measures the rate that a batter reaches base on balls put in play and is computed as follows [17]:

$$\text{DER}=1-\frac{H-HR}{PA-BB-SO-HBP-HR},$$

where *H* = hits, *HR* = home runs, *PA* = plate appearances, *BB* = bases on balls (walks), *SO* = strike outs, and *HBP* = hit by pitches. With the new variable DER, the model in Eq (5) is changed as

$$\begin{array}{c} \ln\left(\pi_{o}\right)=\beta_{1}\ln\left(B_{o}\right)+\beta_{2}\ln\left(P_{o}\right)+\beta_{3}\ln\left(L_{o}\right)+\beta_{4}\ln\left(D_{0}\right). \end{array}$$ (11)

Let *D*_*o*,*i*_ denote the value of *D*_*o*_ for the *i*th matchup, where *D*_*o*_ is the odds ratio of the *DER*: *D*_*o*_ = DER/(1 − DER), let **x**_*i*_ = (ln(*B*_*o*,*i*_), ln(*P*_*o*,*i*_), ln(*L*_*o*,*i*_), ln(*D*_*o*,*i*_))^*T*^ denote a vector of the observations for the *i*th sample, and let ***β*** = (*β*_1_, *β*_2_, *β*_3_, *β*_4_)^*T*^ denote a vector of regression coefficients which *a priori* are from a multivariate normal distribution. Then, the four-variable Bayesian hierarchical log5 model is given by replacing ***β*** = (*β*_1_, *β*_2_, *β*_3_)^*T*^ in the three-variable Bayesian hierarchical log5 model in Section 2.2 with ***β*** = (*β*_1_, *β*_2_, *β*_3_, *β*_4_)^*T*^. In the four-variable Bayesian hierarchical log5 model, ***μ***_0_ is set to ***μ***_0_ = (1, 1, −1, −1)^*T*^ as a pitcher team’s DER is expected to be negatively correlated with the probability of a batter reaching base.

### 4.2 Experimental results

To evaluate whether the newly added variable DER improves the predictive ability of the proposed model, we compared the performance of the four-variable Bayesian hierarchical log5 model with that of competing models: the three-variable Bayesian hierarchical log5 model, the three-variable generalized log5 model (which we considered in Section 3.2), and the four-variable generalized log5 model. The four-variable generalized log5 model was constructed from the model in Eq (11) without the prior distributions over *β*_*i*_, *i* = 1, …, 4. The standard log5 model was not considered as it only contains three variables. The same experimental settings as in Section 3.2 were considered. Table 5 compares the MSE values of the competing models, along with standard errors in parentheses. We can see that the four-variable Bayesian hierarchical log5 model improves upon the three-variable Bayesian hierarchical log5 model and achieves the lowest MSE among the competing models. The performance of the generalized log5 model was also improved when the new variable, DER, was added, although its MSE value was larger than even the three-variable Bayesian hierarchical log5 model. The estimated coefficients were also summarized in Table 5. Similar to Table 2, for the Bayesian hierarchical log5 model, we took an average of the posterior means over all models for different batter/pitcher matchups, and for the generalized log5 model, we took an average of the estimated coefficients over all models for different platoon configurations.

**Table 5:** Comparison between the Bayesian hierarchical log5 models and the generalized log5 models.

	3-var.Bayesian	4-var.Bayesian	3-var.Generalized	4-var. Generalized
**MSE**	0.0464 (0.1522)	0.0428 (0.1389)	0.0780 (0.1939)	0.0515 (0.1597)
*β*_1_	0.8179	0.9761	0.6363	0.9159
*β*_2_	0.1693	0.9284	1.2859	0.8916
*β*_3_	-1.9412	-1.0059	-0.9222	-1.3321
*β*_4_	-	-1.1384	-	-0.2352

### 4.3 Further extension

Recall that the four-variable Bayesian hierarchical log5 model uses the hyperprior mean -1 for the new variable DER based on the fact that DER is negatively related to the probability of a batter reaching base. Although it is reasonable to set a hyperprior mean as a negative value, the specific value -1 is somewhat arbitrary compared to the hyperprior means for other coefficients (i.e., 1, 1, and -1 for *β*_1_, *β*_2_, and *β*_3_, respectively) being set using the coefficients of the standard log5 model. We further extend the four-variable Bayesian hierarchical log5 model by using a different hyperprior mean for ***β***: instead of the coefficients from the standard log5 model, the coefficients of the generalized log5 model are used as a hyperprior mean for ***β***. With the same experimental settings in Section 4.2, we conducted experiments using the four-variable Bayesian hierarchical log5 model with the new hyperprior mean. The results are summarized in Table 6. The coefficients in Table 6 are the average of the posterior means over all models for different combinations of batter and pitcher. Compared with the four-variable Bayesian hierarchical log5 model considered in Section 4.2, the MSE value was obtained to be lower. It may be possible to improve the predictive performance of the Bayesian hierarchical log5 model by trying the two different hyperprior means (i.e., standard log5 model-based, generalized log5 model-based) for training and choosing the one with a smaller training error.

**Table 6:** MSE and the estimated coefficients of the four-variable Bayesian hierarchical log5 model using the coefficients from the generalized log5 model as a hyperprior mean for *β*.

	Bayesian Log5
**MSE**	0.0398 (0.1404)
*β*_1_	0.9909
*β*_2_	0.6583
*β*_3_	-1.5765
*β*_4_	-0.6943

## 5 Conclusion

In this paper, we proposed a new model, the Bayesian hierarchical log5 model, to predict the probability that a batter reaches base when he confronts a particular pitcher using the matchup data of the two players. The Bayesian hierarchical log5 model imposes a prior distribution for the coefficients of the generalized log5 model; the fixed coefficients of the standard log5 model are used to specify the hyperprior for the coefficients. Using a real data example, we showed that the proposed model can predict the probability of a batter reaching base more effectively than the standard log5 model and the generalized log5 model. Unlike the generalized log5 model, the proposed model uses the data of the considered players only; therefore, a more accurate prediction could be achieved when considering a particular batter/pitcher matchup, while overcoming the problem of a lack of available data by using the prior information. The proposed model can be particularly useful when analyzing a matchup between two players in rivalry, for which it would be more desirable to use the data of only the two players to remove the effects of other players. We further extend the proposed model by adding an index for the pitching team’s defense ability, DER. We showed that the extended Bayesian hierarchical log5 model further improves the predictive performance using a real data example. In future research, it may be possible to further extend the proposed model by considering other significant variables. For example, batters’ pitch recognition skills, which are known to be highly related with baseball batting game statistics [18], can be included in the proposed model to further improve the predictive performance.
